# *Shigella flexneri* utilizes intestinal signals to control its virulence

**DOI:** 10.1080/19490976.2023.2256767

**Published:** 2023-09-23

**Authors:** Rimi Chowdhury, Paulina D. Pavinski Bitar, Katherine E. Bell, Craig Altier

**Affiliations:** Department of Population Medicine and Diagnostic Sciences, Cornell University, Ithaca, NY, USA

**Keywords:** Shigella flexneri, Virulence regulation, Intestinal fatty acids, Invasion, Human colon, Enteric pathogens, Intestinal signalling

## Abstract

The enteric pathogens have evolved to utilize elements from their surroundings to optimize their infection strategies. A common mechanism to achieve this is to employ intestinal compounds as signals to control the activity of a master regulator of virulence. *Shigella flexneri* (*S. flexneri*) is a highly infectious entero-invasive pathogen which requires very few organisms to cause invasion of the colonic mucosa. The invasion program is controlled by the virulence master regulator VirF. Here, we show that the fatty acids commonly found in the colon can be exploited by *S. flexneri* to repress its virulence, allowing it to energetically finance its proliferation, thus increasing its pathogenicity. Colonic fatty acids such as oleic, palmitoleic and *cis-*2-hexadecenoic acid were shown to directly bind to VirF and mediate its prompt degradation. These fatty acids also disrupted the ability of VirF to bind to its target DNA, suppressing the transcription of the downstream virulence genes and significantly reducing the invasion of *S. flexneri* to colonic epithelial cells. Treatment with colonic fatty acids significantly increased the growth rate of the pathogen only under invasion-inducing conditions, showing that the reduction in the burden of virulence promotes a growth advantage. These results demonstrate the process by which *S. flexneri* can employ intestinal compounds as signals to increase its numbers at its preferred site of invasion, highlighting the mechanism by which the full spectrum of shigellosis is achieved despite a miniscule infectious dose. This highlights an elegant model of environmental adaption by *S. flexneri* to maximize the pathogenic benefit.

## Introduction

The enteric pathogen *Shigella flexneri* (*S. flexneri*), the causative agent of shigellosis, is a highly infectious human-restricted entero-invasive bacterium.^1–3^ In contrast to other invasive enteric pathogens, *S. flexneri* can cause massive mucosal destruction and inflammatory diarrhea with a remarkably low dose of 10–100 organisms.^4,5^ After ingestion, *S. flexneri* bacteria navigate to the colon, where they are engulfed by the M cells and subsequently by the resident macrophages.^6^ Here, the pathogen causes pyroptotic death of the macrophages and, following release, initiates invasion of the basolateral layer of the colonic epithelium.^7,8^ After invasion, *S. flexneri* dissolves the endocytic vacuole, disseminates, and replicates in the cell cytoplasm. The resulting induction of proinflammatory cytokines and chemokines, leading to extensive inflammatory destruction of the colonic mucosa, is a hallmark of shigellosis.^9,10^

A crucial step in the infectious program of *S. flexneri* is its invasion of the basolateral layer of the colonic epithelium.^8,9^ This is achieved using a molecular apparatus called the Type-3 Secretion System (T3SS).^7,9,11^ The expression of the constituents of this apparatus is initiated by the master virulence regulator encoded by *virF*.^12^ Upon activation, VirF binds to the operator regions of the virulence proteins *virB* and *icsA*, initiating a cascade of activation of downstream invasion genes, leading to the production of the T3SS.^13^ The activation of *icsA* allows the pathogen inter- and intra-cellular motility, an essential part of the infection process of *S. flexneri*.^14,15^ As the production of the T3SS entails the coordinated expression of several virulence genes, it requires an expensive energetic commitment and therefore is tightly regulated by multiple environmental cues at the level of VirF.^15,16^

Several enteric pathogens regulate their virulence programs by employing local compounds from their environment as signals. Long chain fatty acids enriched in the human colon (colonic fatty acids) make an excellent example of such a class of compounds.^17^ Numerous studies have identified and quantified the amounts of long chain fatty acids such as oleic acid, palmitoleic acid, palmitic acid, linoleic acid, etc. in the human feces^18–23^ and found them to be present in millimolar concentrations. *cis*-2-hexadecenoic acid (c2-HDA) is a murine colon constituent,^24^ not yet shown to be present in the human colon. However, this fatty acid is produced by the enzymatic activity of a crotonase encoded by *rpfF*.^25,26^ Numerous bacteria native to the human colonic microbiota harbor *rpfF* such as *Acinetobacter*, *Cronobacter*, *Enterobacter*, *Stenotrophomonas*^27,28^ and thus c2-HDA can be speculated to be human colon constituent.

The colonic fatty acids are utilized by many enteric pathogens, such as *Salmonella*,^24,29,30^ *Vibrio*^31^ and enterotoxigenic *E. coli*,^32^ to regulate their virulence programs. Interestingly, all these pathogens induce their virulence programs by the activation of an AraC-type master regulator, such as HilD, HilC and RtsA of *Salmonella*, ToxT of *Vibrio*, and Rns of enterotoxigenic *E. coli*. It has been shown that many fatty acids control the virulence of these pathogens by directly binding to their respective AraC-type master regulators, which disrupts their ability to bind to their target DNA, thus inhibiting their function as transcriptional activators and preventing the activation of downstream virulence genes^24,29,31–35^. VirF is also an AraC-type protein,^36^ and recently it was shown that certain fatty acids, including diffusible signal factors, can reduce the virulence of *S. flexneri* by binding to VirF.^37,38^ The authors showed that many long chain fatty acids, including palmitoleic acid, can directly prevent the ability of VirF to initiate *virB* transcription and subsequent invasion gene expression. However, the physiological relevance of this interaction to the virulence of *S. flexneri* remains unknown.

Here, we propose that *S. flexneri* must proliferate in the colon to increase its numbers, and hence the likelihood of it being internalized by the M cells, which is essential for successful infection. In this study, we show that *S. flexneri* utilizes the fatty acids found in the colonic milieu, a region preferred by *S. flexneri* for invasion, to repress its virulence and increase its growth rate. This is achieved by the direct binding of these fatty acids such as oleic acid, palmitoleic acid and c2-HDA to VirF, mediating its rapid degradation. This binding also disrupts the ability of VirF to bind to the operator region of its target *virB*, preventing the activation of downstream invasion genes *icsA*, *icsB* and *ipgD*, eventually resulting in significant inhibition of the invasion of colonic epithelial cells. These results highlight the mechanism by which, despite having a low infectious dose, *S. flexneri* can cause the full spectrum of bacillary dysentery.

## Results

### Colonic fatty acids facilitate the growth of *S. flexneri*

**Figure 1.**
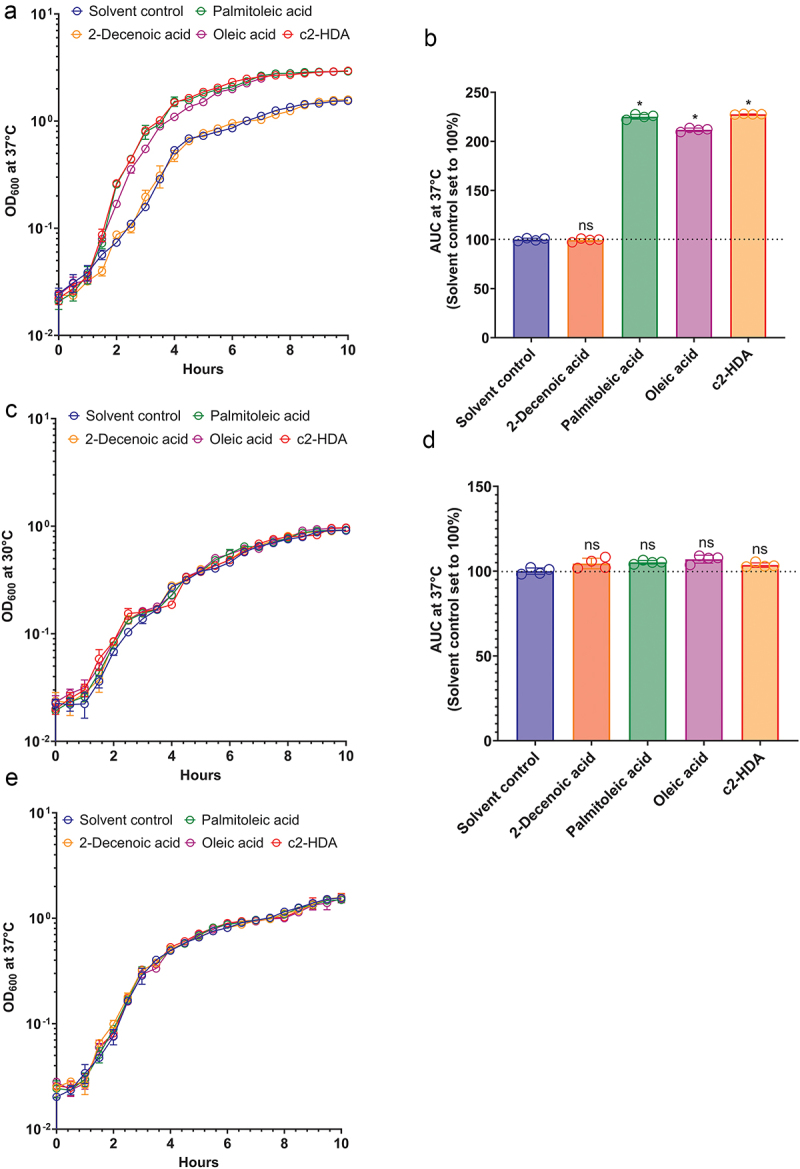
Colonic fatty acids facilitate the growth of *S. flexneri.*

The preferred site of *S. flexneri* invasion, the human colon, is enriched with short- and long-chain fatty acids.^17–22,39^ These fatty acids are utilized by many enteric pathogens as sources of carbon to survive and multiply.^40^ As the infectious dose of *S. flexneri* is less than that of other enteric pathogens, we hypothesized that by utilizing such fatty acids, *S. flexneri* can multiply and increase its numbers in the colon, thus increasing its probability of being engulfed by the colonic M cells. We therefore measured the growth of *S. flexneri* in the presence of various colonic fatty acids at 37°C, a temperature at which virulence functions are known to be induced. Cultures were incubated with 20 µM of the colonic fatty acids c2-HDA, oleic acid or palmitoleic acid, or a medium chain fatty acid *trans*-2-decenoic acid (2-Decenoic acid), not known to be quantified in the human gut. The concentrations of palmitoleic and oleic acids used here are considerably lower than those found in human fecal samples.^18^ We observed a significant increase in growth of *S. flexneri* in the presence of the colonic fatty acids (Figure 1a, b). Treatment with palmitoleic acid or c2-HDA increased the growth rate more than that with oleic acid. The 2-Decenoic acid treated culture grew at a rate similar to that of the solvent control (ethanol) and therefore was used as a control fatty acid for the rest of the study. At 30°C, however, a temperature at which virulence genes are silenced,^41,42^ fatty acid treatment had no significant effect on the growth rate of *S. flexneri* (Figure 1c, d). These results suggest that the fatty acids may increase the growth rate of *S. flexneri* by reducing the burden of virulence. To test this hypothesis, we created a strain lacking the *virF* gene, essential for the pathogenic phenotype of *S. flexneri*^41^ and tested its growth with or without the fatty acid treatment. In the absence of *virF*, the growth rate of *S. flexneri* was not enhanced by the fatty acid treatment at 37°C (Figure 1e). These results show that at physiological temperatures, fatty acids provide a significant growth advantage to the virulent *S. flexneri*, but not to the avirulent. Together, these data suggest that the exposure to such colonic fatty acids may modulate the virulence of *S. flexneri*.

(a, c) Growth of *S. flexneri* cultures treated with solvent control or 20 µM fatty acids was assessed at 37°C (A) or 30°C (C). OD_600_ was measured every half-hour for 10 hours. Data show mean ± standard deviation (SD) of three replicates. (b, d) The area under the curve (AUC) for each treatment at 37°C (B) or 30°C (D) was calculated. The AUC for solvent control at each temperature was set to 100%, and others in the experiment were normalized accordingly. Bars show the normalized AUC ± SD (*n* = 3). Differences between the solvent control and the indicated treatments were calculated by the Mann–Whitney test. *, *P* < 0.05. ns is not significant. (e) Growth of *S. flexneri virF:kan* cultures were treated with solvent control or 20 µM fatty acids and growth was assessed at 37°C. OD_600_ was measured every half-hour for 10 hours. Data show mean ± SD of three replicates.

### Colonic fatty acids reduce the virulence of *S. flexneri*

**Figure 2.**
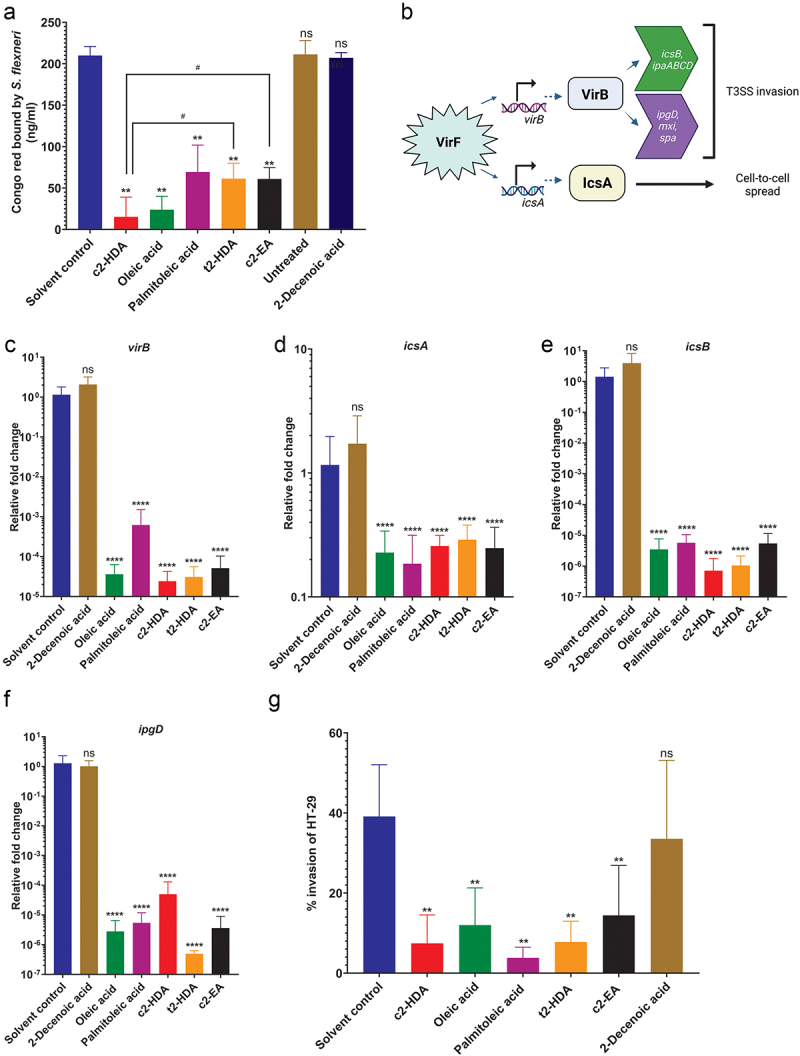
Colonic fatty acids reduce the virulence of *S. flexneri.*

Several fatty acids enriched in the colonic environment are known modulators of virulence gene expression in enteric pathogens.^17^ Therefore, we hypothesized that colonic fatty acids may function to reduce expression of invasion genes in *S. flexneri*. To test this, we performed the widely used Congo red binding assay.^43^ We grew cultures in the presence or absence of 20 µM concentration of fatty acids, added Congo red and assessed the depletion of the dye in the culture media as a measure of bacterial binding capacity. Treatment with c2-HDA resulted in the most severe loss of Congo red binding, indicating a strong repressive effect on the invasion genes, while oleic and palmitoleic acids also significantly reduced binding (Figure 2a). To directly quantify the reduction in invasion gene expression, we measured by RT-qPCR the expressions of *virB*, *icsA*, *icsB* and *ipgD*, genes essential for the function of the T3SS^11^ (Figure 2b). Consistent with the results of the Congo red assay, we found that oleic acid, palmitoleic acid and c2-HDA substantially reduced mRNA levels of these genes, from 5-fold for *icsA* to more than 800-fold for *virB*. (Figure 2c–f). Together, these results demonstrate that colonic fatty acids can strongly repress invasion genes of *S. flexneri*.

(a) Congo red binding by *S. flexneri* cultures treated with solvent control or the fatty acids (20 µM) was assessed. Data show mean ± SD of six replicates. Differences between the solvent control vs. fatty acid treatments were calculated by the Mann–Whitney test. **, *P* < 0.01. Differences between c2-HDA and t2-HDA or c2-EA were calculated by the Mann–Whitney test.^#^, *P* < 0.05. ns is not significant. (b) A simplified illustration of the virulence gene regulation in *S. flexneri*, created with BioRender. (c–f) Relative gene expression of *virB* (C), *icsA* (D), *icsB* (E) and *ipgD* (F) in *S. flexneri* grown with solvent control or indicated fatty acids (20 µM) was measured by RT-qPCR. C_T_ values were normalized to the mean of the housekeeping gene *gapA*. Data show mean ± SD of three biological replicates each having three technical replicates (total *n* = 9). Differences between the solvent control and the indicated treatments were calculated by the Mann–Whitney test. ****, *P* < 0.0001. ns is not significant. G. Invasion of HT-29 cells by *S. flexneri* cultures grown in the presence of solvent control or 50 μM fatty acids was measured by the gentamicin protection assay. Bars show invasion ± SD of two independent experiments with three replicates (total *n* = 6). Differences between the solvent control and the fatty acids were calculated by the Mann–Whitney test. **, *P* < 0.01. ns is not significant.

c2-HDA has been shown to be highly effective in reducing virulence of *Salmonella* and *Vibrio*, and its potency is attributed to its unique structure: 16 carbons in length with a *cis*-unsaturation at second carbon.^24,29,44^ We hypothesized that this distinctive structure may contribute to the increased potency of c2-HDA and therefore compared the efficiency of fatty acids with structures similar to that of c2-HDA in reducing *S. flexneri* virulence. The *trans* stereoisomer of c2-HDA (*trans*-2 hexadecenoic acid or t2-HDA), which differs only in the orientation of the unsaturation at the second carbon, and a longer *cis*-2 fatty acid (20 carbons), *cis*-2 eicosenoic acid (c2-EA), reduced *S. flexneri* Congo red binding by ~ 3-fold, significantly less reduction than that of c2-HDA. These results thus show that the *cis*-2 unsaturation and the 16-carbon structure of c2-HDA play important roles in the efficacy of this chemical.

Next, we investigated the effect of these compounds on *S. flexneri* invasion by testing them in invasion assays. We used the colonic epithelial cell line HT-29 and performed gentamicin protection assays with cultures treated with or without the fatty acids. We found that treatment with c2-HDA drastically repressed *S. flexneri* invasion when supplied at a concentration of 50 µM. The *trans* isomer t2-HDA and the 20-carbon c2-EA had a similar effect, repressing invasion significantly. Treatment with oleic or palmitoleic acid substantially reduced the invasion ability of *S. flexneri* (Figure 2g). These results thus demonstrate that fatty acids of varying classes, including those found in the colon, can remarkably restrict the invasion gene expression of *S. flexneri*, with differing efficiencies.

### Colonic fatty acids function through VirF to inhibit the Congo red binding

**Figure 3.**
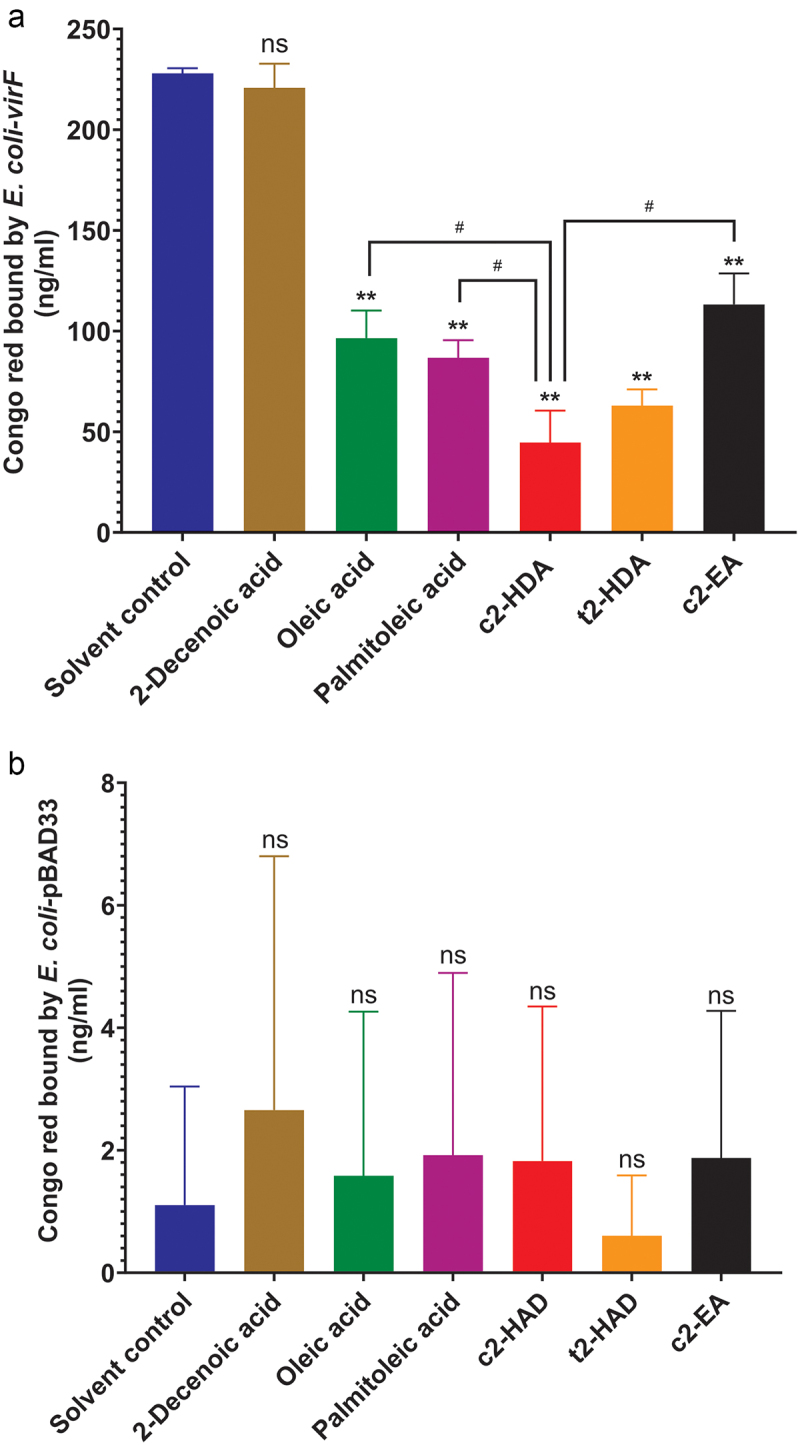
Colonic fatty acids function through VirF to inhibit the Congo red binding.

The transcription of *virB* is activated by the master transcriptional regulator of *S. flexneri* invasion VirF. To test the role of *virF*, we placed this gene under an arabinose-inducible promoter on a plasmid in *E. coli*. Previous studies have demonstrated that *virF* alone is sufficient to impart the ability to bind Congo red to *E. coli*,^45^ and therefore we used the same quantitative Congo red binding assay to assess the effects of fatty acids due solely to *virF*. As expected, upon arabinose induction, *E. coli* expressing *virF* efficiently bound Congo red (Figure 3a). Consistent with the results obtained for *S. flexneri*, Congo red binding was significantly diminished by all fatty acids previously tested, with c2-HDA being the most effective. The *trans* isomer t2-HDA and the 20-carbon fatty acid c2-EA also showed significant effects but were less efficient than c2-HDA. The control 2-Decenoic acid did not prevent the binding of Congo red, showing that the binding of the dye is not due to the nonspecific membrane interactions, thus demonstrating the specificity of the assay. As Congo red binding may be influenced by other factors as well, we performed this assay using an *E. coli* strain with the control vector pBAD33 lacking *virF* (Figure 3b). We did not observe any notable differences in Congo red binding due to the fatty acid treatment, showing the specificity of our findings. These results establish that colonic fatty acids act to inhibit the function of *virF* and that specific structural constituents of these molecules are essential to their efficiency.

(a, b) Congo red binding by *E. coli* cultures having plasmid with arabinose-inducible *virF* (a) or empty vector (b), treated with solvent control or indicated fatty acids (20 µM). Data show mean ± SD of six replicates. Differences between the solvent control and the fatty acid treatments were calculated by the Mann–Whitney test. **, *P* < 0.01. ns is not significant. Differences between c2-HDA and indicated fatty acid treatments were calculated by Mann Whitney test.^#^, *P* < 0.05.

### Colonic fatty acids act by binding directly to VirF

**Figure 4.**
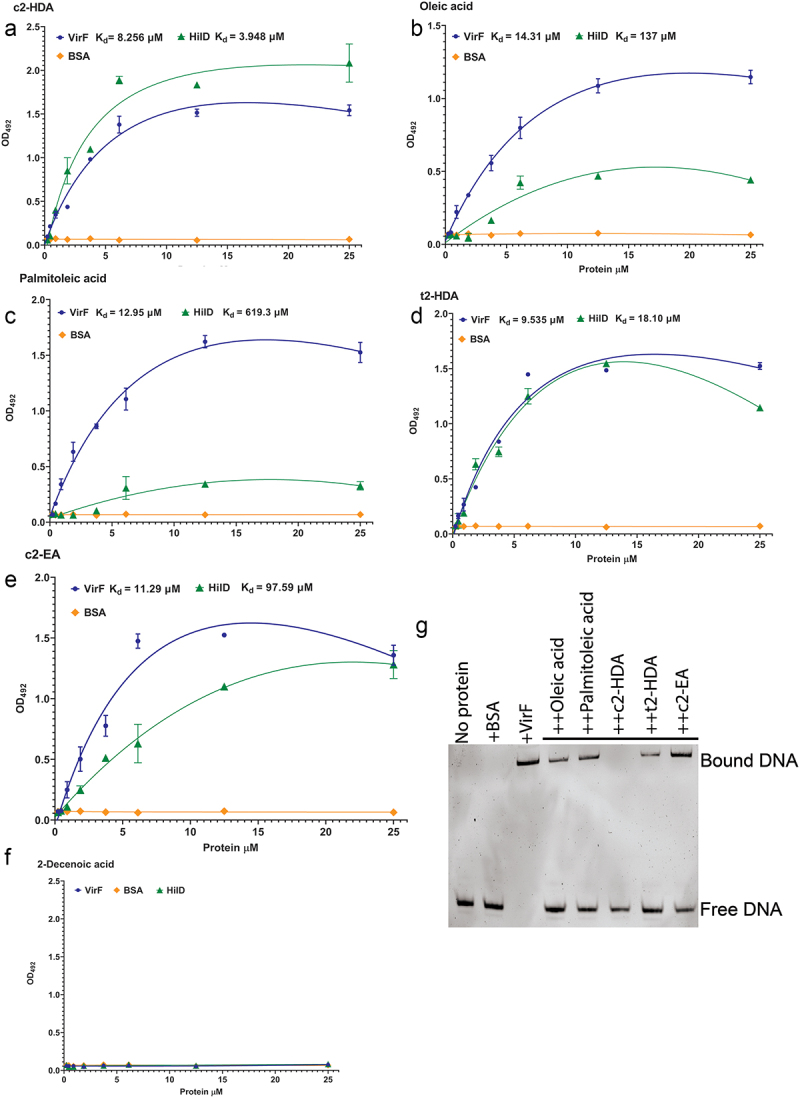
Colonic fatty acids act by binding directly to VirF.

The results presented here demonstrate that colonic fatty acids impair the function of *virF*, but do not implicate a specific mechanism of action. It is known that colonic fatty acids such as c2-HDA and palmitoleic acid bind to AraC-type transcription regulators in *Salmonella, Shigella* and *Vibrio* to repress virulence.^24,33,38^ As VirF is also an AraC-type transcriptional regulator, and recently it was shown that VirF can interact with some fatty acids directly,^38^ we hypothesized that colonic fatty acids may repress VirF through direct interaction. To test this, we expressed and purified VirF protein and evaluated its binding to fatty acids using an ELISA assay. Wells of polystyrene plates were coated with each fatty acid and various concentrations of His-tagged VirF were added and allowed to bind. Binding was detected using an anti-His antibody, and binding curves with nonlinear regression were plotted to determine the dissociation constants (K_d_). c2-HDA showed the highest affinity for VirF with an apparent K_d_ of 8.2 µM, followed by palmitoleic acid (12.9 µM) (Figure 4a, b). Oleic acid (14.3 µM) showed strong binding but less than that of c2-HDA (Figure 4c). BSA demonstrated no binding to any of the fatty acids, showing the specificity of the assay. As c2-HDA showed the highest binding affinity among these fatty acids, we assessed the importance of the *cis*-2 unsaturation on VirF binding by testing variants of c2-HDA, finding that the *trans* stereoisomer t2-HDA and the *cis*-2 fatty acid with 20 carbons, c2-EA, did not bind as efficiently to VirF as did c2-HDA (9.5 and 11.2 µM, respectively) (Figure 4d, e). The control 2-Decenoic acid did not show any binding to VirF demonstrating the specificity of the binding assays (Figure 4f).

(a–f) Increasing concentrations of HilD or VirF or BSA were added to 96-well plates pre-adsorbed with 100 μM of indicated fatty acids. Binding was detected by anti-His antibody. Non-linear regression was plotted to calculate K_d_. G. EMSA of *virB* promoter DNA (100 nM) in the presence of 250 μM BSA or VirF with 40 µM concentration of indicated fatty acids.

VirF directly activates transcription of *virB* and *icsA* by binding to their promoter DNA, initiating the invasion program of *S. flexneri*. We determined next whether binding of these fatty acids to VirF prevented its ability to bind to its target DNA. For this, we employed electrophoretic mobility shift assays (EMSA), incubating *virB* promoter DNA with the VirF protein, with or without fatty acids. We found that the addition of 40 µM oleic acid or palmitoleic acid substantially disrupted this binding (Figure 4g). Interestingly, at this concentration, c2-HDA was the only fatty acid to completely disrupt the VirF-P*virB* complex. Together, these results show that these colonic fatty acids can directly bind to VirF with high affinity and disrupt its binding to the *virB* promoter, thus defining the mechanism by which they can prevent the activation of the T3SS.

### *Colonic fatty acids mediate rapid degradation of VirF in* S. flexneri

**Figure 5.**
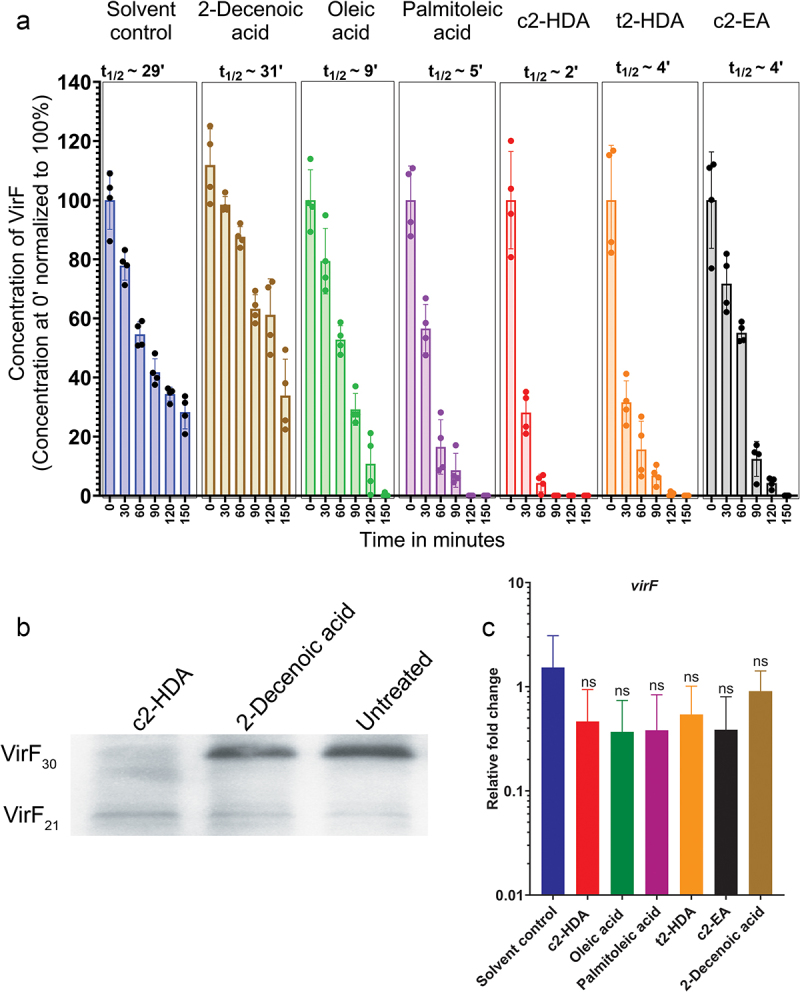
Colonic fatty acids mediate rapid degradation of VirF in *S. flexneri*.

The colonic fatty acid c2-HDA can directly bind to the AraC-type protein HilD in *Salmonella* and mediate its rapid degradation.^24^ We thus investigated whether the binding of c2-HDA similarly led to the degradation of VirF. To test this, we constructed a *S. flexneri virF* mutant strain complemented with a plasmid carrying *virF* under the control of a tetracycline-inducible promoter and with a C-terminal 3XFLAG tag. Cultures grown with or without fatty acids were induced with tetracycline to initiate VirF production. To standardize the number of cells and subsequently total protein, cultures were grown to OD_600_ of 1, and the production of new protein was halted by adding an antibiotic cocktail. We tracked the stability of VirF by collecting aliquots at various timepoints and quantified the amount of VirF by an anti-FLAG tag ELISA. We observed that, VirF half-life was severely reduced by c2-HDA treatment (t_1/2_ ~2 minutes) (Figure 5a). t2-HDA and c2-EA also reduced VirF lifetime but less efficiently than did c2-HDA. Treatment with oleic acid or palmitoleic acid also reduced VirF lifetime similarly (t_1/2_ ~9 minutes and ~5 minutes, respectively). The *virF* ORF can produce two forms of VirF: VirF_30_ (30 kD) and VirF_21_ (21 kD) by differential translation.^46,47^ Therefore, we investigated whether fatty acids mediated the degradation of both these forms of VirF. We observed that VirF_30_ was rapidly degraded by the exposure to c2-HDA (Figure 5b). However, the levels of VirF_21_ remained similar, which demonstrates that these fatty acids may preferentially mediate degradation of only VirF_30_. In addition, these fatty acids did not affect *virF* mRNA levels (Figure 5c), showing that the effect is not transcriptional and confirming that they modulate VirF_30_ protein levels. These results thus demonstrate that specific structural moieties of repressive fatty acids directly contribute to their efficiency at controlling VirF lifetime. Together, these results highlight an important mechanism by which intestinal compounds are utilized by enteric pathogens to regulate their virulence.

(a) *S. flexneri virF:kan* strains containing a tetracycline-inducible *virF*-3XFLAG plasmid were grown to log phase in solvent control or 20 µM of indicated fatty acids. Expression of *virF* was induced with 5 µg/ml tetracycline. Transcription and translation were halted by addition of an antibiotic cocktail, and the protein half-life was assessed by quantification of VirF by anti-FLAG tag ELISA. FLAG tagged protein concentration at 0 minutes was set to 100% and other time-points were normalized accordingly. Bars show normalized mean concentration ± SD of four independent experiments. Individual lifetimes from all experiments were averaged to calculate the reported half-life values. (b) Western blot of *S. flexneri virF* mutant strain complemented with plasmid encoding VirF-3XFLAG under tetracycline promoter. Cultures were induced with 5 µg/ml tetracycline and treated with or without 20 µM of indicated fatty acids. VirF was probed using an anti-FLAG antibody. (c) RT-qPCR showing *virF* mRNA levels in *S. flexneri* strains treated with 20 µM of indicated fatty acids.

## Discussion

The infectious dose of *S. flexneri* is exceedingly low compared to other enteric pathogens. As few as 10–100 microorganisms are sufficient to cause disease.^4^ However, the mechanism by which *S. flexneri* can produce disease with just a few organisms has remained unexplored. In this study, we show that *S. flexneri* can utilize colonic long-chain fatty acids to enhance its growth rate by reducing its virulence burden. This growth advantage is achieved through the control of VirF: colonic fatty acids directly bind to this master virulence regulator, both preventing its binding to its target *virB* operator DNA and inducing its own rapid degradation. Together, these lead to extensive reduction in the expression of downstream invasion genes *virB*, *icsA*, *icsB* and *ipgD* and significantly reduce invasion of colonic epithelial cells. Our results demonstrate that, by this mechanism, *S. flexneri* may utilize compounds enriched in the colonic milieu to overcome its virulence burden and thus facilitate its growth for its pathogenic benefit.

Engulfment by the M cells is crucial for *S. flexneri* to cause disease. Capture by these cells leads to rapid phagocytosis by the resident macrophages, where *S. flexneri* causes pyroptosis and, following release, induces invasion of the basolateral layer of the colonic epithelium. This leads to initiation of the full spectrum of inflammation and subsequent disease phenotype.^48^ Our results show that *S. flexneri* can use colonic fatty acids to increase its numbers. Thus, we hypothesize that the success of *S. flexneri* infection depends upon its utilization of the colonic fatty acid so that it can be engulfed in greater numbers by the M cells. Additionally, it is also reasonable to speculate that *S. flexneri* may have evolved to preferentially invade the colon because of the enrichment of such fatty acids in the colonic milieu.

VirF presumably has a distinct binding pocket for fatty acid attachment, as has been shown for AraC-type transcriptional activators of other bacterial species,^31,32^ and this putative pocket may have specificity toward certain conformations of fatty acids. We found that different colonic fatty acids bind to VirF with different efficiencies; c2-HDA bound with relatively higher affinity than did oleic or palmitoleic acids (Figure 4). In comparison, increasing the carbon tail length or changing the orientation of the *cis*-unsaturation at the second carbon to *trans-* reduced the binding affinity of the fatty acid. Supporting these data, a recent report by Trirocco et al.^37^ also showed that binding of VirF to *virB* promoter DNA was reduced in the presence of medium- and long-chain fatty acids, with long-chain unsaturated fatty acids showing higher effectiveness. The authors predict that VirF has a distinct binding “cleft” which can accommodate saturated fatty acids in an extended conformation using the amino acid H212. In contrast, fatty acids such as palmitoleic acid may adopt different conformations around their unsaturation and thus may employ additional amino acids such as H17 and K216 to bind. This may also explain the mechanism by which 2-Decenoic acid failed to bind to VirF, probably because of the shorter length of its C-tail. Further structural analysis of VirF may elucidate how this binding pocket can accommodate fatty acids of diverse conformations with different affinities.

Our experiments show that VirF lifetime is extensively reduced upon interaction with colonic fatty acids. A recent report by Xerri et al.^49^ similarly showed that VirF levels are reduced by exposure to the lipids present in the outer membrane vesicles of *B. thetaiotaomicron*. The AraC-type master regulator of *Salmonella* invasion, HilD is also rapidly degraded upon interaction with fatty acids.^24^ Such post-translational control of regulatory AraC-type proteins is a common mechanism as it leads to prompt and efficient silencing of invasion genes controlled by this regulator. In contrast, a recent report found that treatment with palmitoleic acid did not lead to decreased levels of VirF.^37^ This discrepancy can be attributed to the fact that in our experiments, after treatment with fatty acid the production of new protein was halted by adding an antibiotic cocktail. This is important because as cells grow, they produce new protein, diluting the effect of the treatment which might lead to such incongruities. Nonetheless, it will be interesting to see if such a post-translational control mechanism is used by several enteric pathogens that have AraC-type master regulators, or if this is unique to *Salmonella* and *S. flexneri*.

## Materials and methods

### Bacterial strains and growth conditions

All the bacterial strains and plasmids used in this study are listed in Table 1. *S. flexneri* was maintained in trypticase soy broth (TSB) and TSB agar grown with 0.01% Congo red dye at 37°C. Strains of *S. flexneri* producing white colonies on Congo red agar plates and negative for *virF* amplification using PCR were considered avirulent.^41,50^ *E. coli* strains were maintained in Luria-Bertani (LB) broth (1% Tryptone, 0.5% yeast extract, 0.5% NaCl) and LB with 1.5% agar. All liquid cultures were grown in TSB or LB broth, aerobically with continuous shaking (200 rpm) at 37°C or 30°C incubator, unless mentioned otherwise. Growth was measured by recording OD600 of aliquots of growing cultures. All fatty acids were purchased from Cayman chemicals. For each experiment, a range of concentrations for each fatty acid were performed to identify which concentrations showed their maximum efficacy. This method helped us analyze which fatty acids are more potent compared to others in each assay.

**Table 1.** Strains and plasmids used in the study.

**Strain**	**Description**	**Source**
CA216	Wild type S. flexneri 2457T	P. Orndorff laboratory
CA12	E. coli MG1655	Mudasir et al
CA5318	E. coli pBAD33-virF or E. coli-virF	This study
CA5319	E. coli-pBAD33	This study
CA5712	S. flexneri virF::kan	This study
CA5720	S. flexneri virF::kan pRC5576	This study
CA5324	E. coli pET15b-virF	This study
**Plasmid**	**Description**	**Source**
pRC5585	pBAD33-virF	This study
pRC5576	pWSK29-tetRA-virF-3XFLAG	This study
pRC5587	pET15b-virF	This study

### Construction of *S.* *flexneri virF* mutant

This was performed as described previously.^51^ Using plasmid pKD4, the DNA region encoding resistance against kanamycin was amplified by PCR using primers virFDW FP/RP having a 40-bp homology extension flanking *virF*. The PCR fragment was electroporated into a strain expressing λ Red recombinase. Loss of *virF* was confirmed by PCR.

### Construction of *E. coli-virF*

The open reading frame of *virF* from the genome sequence of *S. flexneri* 2457T was cloned using primers virFpBAD33 FP/RP and pBAD33 FP/RP into pBAD33 by Gibson assembly.^52^ Clones were confirmed by PCR using primers pBAD33chk FP/RP and by DNA sequencing. Plasmids were isolated from successful clones and electroporated into *E. coli* MG1655. *virF* expression was induced by adding 0.2% arabinose to growing cultures.

### Growth assessment assay

Overnight cultures of *S. flexneri* were diluted in 5 ml TSB with 100 mM MOPS [3-(N-morpholino) propanesulfonic acid)] to a final OD_600_ of 0.02. These were treated with ethanol (solvent control) or 20 µM concentration of fatty acids dissolved in ethanol. Cultures were grown in 15 ml glass tubes for 10 hours at 37°C or 30°C with continuous shaking at 200 rpm. Aliquots of 200 µl were taken out and growth was measured by checking OD_600_ every half-hour in a spectrophotometer. Growth curves were plotted on a semi-log scale in GraphPad Prism.

### Congo red binding assay

These were performed as described in Qadri et al.^43^ *E. coli* strains having the empty vector pBAD33 or having *virF* under arabinose inducible promoter (*E. coli-virF*) or *S. flexneri* cultures treated with solvent control or fatty acids, were grown to OD_600_ of 1. All fatty acids were used at 20 µM concentration. In case of *E. coli-virF*, expression was induced by adding 0.2% arabinose to the growth medium. After growth, culture volume of 1 ml was centrifuged and washed once with 1 ml of sterile PBS (137 mM NaCl, 2.7 mM KCl, 8 mM Na_2_HPO4, 2 mM KH_2_PO4, pH 7.5). Pellets were resuspended in 1 ml PBS containing 50 µg of Congo red and incubated in the dark at room temperature for 10 minutes. Cells were centrifuged and the unbound dye remaining in the supernatant was quantified spectrophotometrically at 480 nm. PBS with Congo red was used as control and depletion of the dye because of binding by the bacteria was calculated accordingly. Congo red bound by the solvent control treated culture was considered 100% and others were calculated accordingly.

**Table 2.** Primers used in the study.

**Primer name**	**Primer sequence**
virFDW FP	5’- CTGTAAACACTAAATATAGTTTGGTATATTCTGTTGAATTTGATTGTGTAGGCTGGAGCTGCTTCGAAGTTCCTAT-3’
virFDW RP	5’-AAGTAAAATTTCTTTGGAGTTATACCATAATATTCATTAACCATGGTCCATATGAATATCCTCCTTAGTTC-3’
pBAD33 FP	5’- TATATCATAAAAAATTTTAAATGCAAGCTTGGCTGTTTTGG-3’
pBAD33 RP	5’- TTATGTCCCATATCCATCATCCGAGCTCGAATTCGCTAGC-3’
virFpBAD33 FP	5’- GCTAGCGAATTCGAGCTCGGATGATGGATATGGGACATAAAAACAAAATAGATATAAAGGT-3’
virFpBAD33 RP	5’-CAAAACAGCCAAGCTTGCATTTAAAATTTTTTATGATATAAGTAAAATTTCTTTGGAGTTATACCA-3’
pBAD33chk FP	5’-CAACTCTCTACTGTTTCTCCA-3’
pBAD33chk RP	5’- TGATTTAATCTGTATCAGGCT-3’
pet15 FP	5’-TTATGTCCCATATCCATCATGCTGCCG-3’
pet15 RP	5’-ACTTATATCATAAAAAATTTGGCTGCTAACAAAGCCCG-3’
virFpet15 FP	5’-TTCGGGCTTTGTTAGCAGCCAAATTTTTTATGATATAAGTAAAATTTCTTTGGAGTTA-3’
virFpet15 RP	5’-GCCTGGTGCCGCGCGGCAGCATGATGGATATGGGACATAAAAACAAAATAGATATAA-3’
pET15bchk FP	5’-ATGCGGTAGTTTATCACAGTTAAA-3’
pET15bchk RP	5’- TAATACGACTCACTATAGGGGAAT-3’
virFpWSK29 FP	5’-AATTTTGATTTACACGCACATTTAATAAAAGGAAATTATCATGATGGATATGGGACATAAAAACA-3’
virFpWSK29 RP	5’-GATATCATGATCTTTATAATCACCGTCATGGTCTTTGTAGTCAAATTTTTTATGATATAAGTAAAATTTC-3’
pWSK29 set1 FP	5’-GGTATAACTCCAAAGAAATTTTACTTATATCATAAAAAATTTGACTACAAAGACCATGACGGTGATTATAAAGATC-3’
pWSK29 set1 RP	5’-CCTTTATATCTATTTTGTTTTTATGTCCCATATCCATCATGATAATTTCCTTTTATTAAATGTGCGTGTAAATCAAAATTATTC-3’
pWSK29 set 2 FP	5’-CCAAAAACTCGTAAAAGCTCTGATGTATCTATCTTTTTTACACCGTTTTC-3’
pWSK29 set 2 RP	5’-CGCTTCCTCGCTCACTGACTCGCTGCGCTCGGTCGTTCGGCT-3’
pWSK29 set 3 FP	5’-GCAGCGAGTCAGTGAGCGAGGAAGCGGAAGAGCGCCCAATA-3’
pWSK29 set 3 RP	5’-ATAGATACATCAGAGCTTTTACGAGTTTTTGGTGCATTCAAAGCTGTTCAC-3’
virBpro FP	5’-ATCACACCCTGTTTATTCATA-3’
virBpro RP	5’-AATTGATAAGCATTTTTTCATCTATGGAGC-3’
virBRT FP	5’-CGAATGTACGCGATCAAGAATCCC-3’
virBRT RP	5’-GATGCTCTTCTACGAGTGCCATCC-3’
icsART FP	5’-GGCGACAGTCATGGAGAGTTCAAT
icsART RP	5’-CCAAAATGAGAGTTCCCTCCCCTG-3’
icsBRT FP	5’-GCCGCCTATGAAGCAGCTAAAAAC-3’
icsBRT RP	5’-GGGTTATGCCTCTATTTCCCCAGC-3’
ipgDRT FP	5’-AAGCTGAGGAGTTAGTAAGCGCAG-3’
ipgDRT RP	5’-TACTCTCCTCTCCCCCGGTTAAAC-3’
virFRT FP	5’-TCTGAGGAGAAAAGGGGGTTAAACA-3’
virFRT RP	5’- AAGCTTCCTCATCAGAAACAGCTG-3’
gapART FP	5’-TGAAGCAACTGGTCTGTTCCTGAC-3’
gapART RP	5’-AACGATGTCCTGGCCAGCATATTT-3’

### Expression of HilD and VirF

Protein expression was performed as described previously.^24,29,30^ The open reading frame of *virF* was cloned into pET15b using primers virFpet15 FP/RP and pET15 FP/RP listed in Table 2, by Gibson assembly. Clones were confirmed by PCR using primers pET15bchk FP/RP and DNA sequencing. Plasmids were electroporated into *E. coli* BL21 DE3 for expression. Cultures were grown to OD_600_ of 0.6 and induced with 5 mM IPTG (Isopropyl-β-D-1-thiogalactopyranoside) and grown for four hours aerobically at 37°C. Cells were centrifuged and aliquots were run on 12% SDS-PAGE to confirm induction of protein expression. Post confirmation, pellets were dissolved in Xtractor buffer followed by sonication to lyse the cells. Talon beads (TaKaRa) were used to extract His-tagged VirF by affinity purification following Takara’s protocol. Elution of VirF was confirmed by SDS-PAGE. Eluted proteins were dialyzed overnight in dialysis buffer (50 mM sodium phosphate, 300 mM NaCl, 10% glycerol). Size and purity of the protein were confirmed by running aliquots on 12% SDS-PAGE. Protein concentrations were calculated in NanoDrop (Thermo Scientific).

### ELISA

These were performed as previously described in Chowdhury et al.^24^ 96-well polystyrene plates were coated with 100 µM fatty acids in coating buffer (0.05 M sodium carbonate-bicarbonate, pH 9.3) and stored overnight at 4°C. Wells were rinsed with sterile PBS and blocked for two hours with 100 µl of blocking buffer (1% Ficoll 400 in PBS) at room temperature. Wells were washed three times with 200 µl PBS and different concentrations of VirF were added and incubated at room temperature for two hours. After incubation, wells were washed three times with 200 µl PBS to remove unbound protein. Wells were incubated with 100 µl of anti-His tag antibody (1:1000 dilution in blocking buffer) and incubated at room temperature for one hour. Unbound antibody was washed off and wells were incubated with 100 µl of secondary anti-mouse antibody (1:5000 dilution in blocking buffer) at room temperature for one hour. After incubation, wells were washed three times with 200 µl wash buffer (PBS-0.1% tween 20) and 100 µl of OPD substrate was added. Reaction was stopped by adding 50 µl stop solution (1N sulfuric acid). The color was read at 490 nm in a spectrophotometer and binding was evaluated in GraphPad Prism.

### EMSA

These were performed as described by Chowdhury et al. in 2021^24,29^ with modifications. *virB* promoter^53^ was amplified using primers virBpro FP/RP stated in Table 2 and purified by Qiagen PCR cleanup kit following their protocol. *virB* promoter (100 nM) was incubated with 250 µM VirF with or without 40 µM concentration of fatty acids in binding buffer (10 mM Tris pH 7.5, 1 mM EDTA, 100 mM KCl, 0.1 mM DTT, 5% v/v glycerol, 0.01 mg/ml BSA). The binding reaction was performed for 15 minutes in a 37°C water-bath. 10X dye solution (10 mM Tris pH 7.5, 1 mM EDTA, 50% v/v glycerol, 0.001% w/v bromophenol blue) was added to a final concentration of 1X. A 6% polyacrylamide gel was prepared, and a pre-run was performed for one hour at 100 V in 1X TAE buffer (40 mM Tris, 20 mM acetic acid, 2.5 mM EDTA, pH 9.5). Samples were loaded and electrophoresis was performed at 100 V for two hours. Post run gels were immersed in 1X SYBR green and bands were visualized in BioRad ChemiDoc Imaging system.

### Gentamicin protection assay

These were performed as described previously^24,29,54,55^ with modifications. *S. flexneri* cultures were grown with or without 50 µM concentration of fatty acids to OD_600_ of 1 in TSB. About 5 × 10^7^ CFU diluted in PBS were added to monolayers of colonic epithelial cell line HT-29 at a multiplicity of infection of 100. Plates were centrifuged at 500 × *g* for 10 min for synchronization and incubated for 30 min at 37°C incubator with 5% CO_2_. After incubation, wells were washed three times with PBS to remove external bacteria. Wells were incubated with media containing 200 µg/ml gentamicin for 1 hour in a 37°C incubator with 5% CO_2_. Wells were then washed three times with PBS and lysed with 0.1% Triton X-100 to recover intracellular bacteria. Serial dilutions of both the inoculum and the lysate were plated on TSA plates. Invasion rate of each sample was calculated using this formula: percent invasion = 100% x [(CFU in lysate X dilution factor)/CFU in inoculum].

### RNA isolation, cDNA synthesis and RT-qPCR

Overnight cultures of *S. flexneri* were diluted 1:100 in TSB with or without 20 µM concentration of fatty acids and grown aerobically at 37°C for four hours. Total bacteria were collected by centrifugation at 10,000 rpm for 5 minutes. Supernatant was discarded and the pellet was resuspended in 1 ml of TRIzol (Invitrogen) and incubated for five minutes at room temperature. RNA was extracted by following manufacturer’s protocol and TURBO DNAse (Invitrogen) was used to remove contaminating DNA. iTaq Universal SYBR Green One-Step Kit (Bio-Rad) was used to convert the RNA to cDNA and carry out RT-qPCR in a single reaction mix using primers gapART FP/RP, virBRT FP/RP, icsART FP/RP, icsBRT FP/RT, ipgDRT FP/RP and virFRT FP/RP, following the manufacturer’s protocol for MiSeq users. Relative expression of *virB*, *icsA*, *icsB* and *ipgD* were calculated by using the threshold cycle (∆∆C_t_) method^56^ relative to the housekeeping gene *gapA*.

### Degradation assay

The half-life of VirF/HilD was assessed as described in Chowdhury et al.^24^ with alterations. The plasmid pWSK29-*tetRA-virF-3×FLAG* was constructed by Gibson assembly using primers virFpWSK29 FP/RP, pWSK29 set1 FP/RP, pWSK29 set 2 FP/RP and pWSK29 set 3 FP/RP, listed in Table 2. *S. flexneri virF:kan* strains were transformed with the plasmid pWSK29-*tetRA-virF-3×FLAG*. These were grown overnight and then diluted 1:100 into LB with appropriate antibiotics and 20 µM fatty acids or solvent control. VirF expression was initiated by adding 5 µg/ml tetracycline. These were grown until OD_600_ of 1 to confirm equal density of cultures at the half-life assay starting point. New protein production was halted by the addition of a cocktail of antibiotics (100 µg/ml rifampin, 200 µg/ml streptomycin and 50 µg/ml spectinomycin). Cultures were then incubated at 37°C and 200 µl aliquots were collected at every half-hour time-point. Bacteria was collected by centrifugation and lysed by freeze-thaw. Lysates were added to FLAG antibody pre-coated 96-well plates (ABSbio DYKDDDDK tag ELISA kit SE002) and incubated at room temperature for two hours. The amount of FLAG-tagged VirF present in the lysate was quantified by ELISA as per manufacturer’s protocol. The quantity of VirF present at each time-point was calculated from a standard curve prepared by using different dilutions of a FLAG fusion protein. The half-life was calculated from the difference between the first time point (0 minutes) and the last time point at which signal could be detected, using the following equation: t_1/2_ = (t x ln2)/[ln(N_0_/N_f_)], where t_1/2_ is the half-life (in minutes), t is the time elapsed between time-points, N_0_ is the concentration obtained at first time-point, and N_f_ is the concentration obtained at the last time-point.

### Estimation of VirF_30_ and VirF_21_

*S. flexneri virF::kan* strains having pWSK29-*tetRA-virF-3XFLAG* were grown with or without the indicated fatty acids at 20 µM concentration until OD_600_ of 1. New protein production was halted by the addition of a cocktail of antibiotics (100 µg/ml rifampin, 200 µg/ml streptomycin and 50 µg/ml spectinomycin). These are grown for three hours and samples were aliquoted and precipitated. Bacterial pellets were lysed by adding laemmli buffer. Proteins were separated on 12% acrylamide gels by SDS-PAGE and the relative levels of VirF_30_ and VirF_21_ were analyzed in western blot by using anti-FLAG antibodies.

### Statistical analysis

All experiments were performed at least three times with the data shown being representative of at least one experiment (mean ± SD). VirF binding constants (K_d_ values) with different fatty acids were determined by nonlinear curve fitting using GraphPad Prism. The Mann – Whitney test was performed to compare means of fatty acid and solvent control treated samples in GraphPad Prism. Differences were considered significant when *P* < 0.05 (indicated by^#^), *P* < 0.01 (indicated by **) and *P* < 0.0001 (indicated by ****).

## Data Availability

All relevant data can be found here: CHOWDHURY, RIMI (2023), “Shigella flexneri utilizes intestinal signals to control its virulence.”, Mendeley Data, V1, doi: 10.17632/stfz3ztdwt.1
